# Na^+^K^+^-ATPase Activity and K^+^ Channels Differently Contribute to Vascular Relaxation in Male and Female Rats

**DOI:** 10.1371/journal.pone.0106345

**Published:** 2014-09-04

**Authors:** Fernanda Moura Vargas Dias, Eduardo Hertel Ribeiro, Aurélia Araújo Fernandes, Jonaina Fiorim, Teresa Cristina Francischetto Travaglia, Dalton Valentim Vassallo, Ivanita Stefanon

**Affiliations:** Universidade Federal do Espírito Santo, Departamento de Ciências Fisiológicas, Vitória, Espírito Santo, Brasil; University Hospital of Würzburg, Germany

## Abstract

Gender associated differences in vascular reactivity regulation might contribute to the low incidence of cardiovascular disease in women. Cardiovascular protection is suggested to depend on female sex hormones’ effects on endothelial function and vascular tone regulation. We tested the hypothesis that potassium (K^+^) channels and Na^+^K^+^-ATPase may be involved in the gender-based vascular reactivity differences. Aortic rings from female and male rats were used to examine the involvement of K^+^ channels and Na^+^K^+^-ATPase in vascular reactivity. Acetylcholine (ACh)-induced relaxation was analyzed in the presence of L-NAME (100 µM) and the following K^+^ channels blockers: tetraethylammonium (TEA, 2 mM), 4-aminopyridine (4-AP, 5 mM), iberiotoxin (IbTX, 30 nM), apamin (0.5 µM) and charybdotoxin (ChTX, 0.1 µM). The ACh-induced relaxation sensitivity was greater in the female group. After incubation with 4-AP the ACh-dependent relaxation was reduced in both groups. However, the dAUC was greater in males, suggesting that the voltage-dependent K^+^ channel (*K_v_*) participates more in males. Inhibition of the three types of Ca^2+^-activated K^+^ channels induced a greater reduction in *R_max_* in females than in males. The functional activity of the Na^+^K^+^-ATPase was evaluated by KCl-induced relaxation after L-NAME and OUAincubation. OUA reduced K^+^-induced relaxation in female and male groups, however, it was greater in males, suggesting a greater Na^+^K^+^-ATPase functional activity. L-NAME reduced K^+^-induced relaxation only in the female group, suggesting that nitric oxide (NO) participates more in their functional Na^+^K^+^-ATPase activity. These results suggest that the K^+^ channels involved in the gender-based vascular relaxation differences are the large conductance Ca^2+^-activated K^+^ channels (*BK_Ca_*) in females and *K_v_* in males and in the K^+^-induced relaxation and the Na^+^K^+^-ATPase vascular functional activity is greater in males.

## Introduction

Gender-associated differences in the development of cardiovascular diseases have been described in humans and animals [1]–[3]. These differences in vascular reactivity regulation could explain the low incidence of cardiovascular disease in women in the reproductive period, such as stroke, hypertension and atherosclerosis [4], [5]. The cardiovascular protection observed in females has been attributed to the beneficial effects of estrogen on endothelial function [6]. The hormone 17ß-estradiol is a potent stimulus for endothelial nitric oxide synthase (eNOS) activation, and NO release [7]–[10]. NO is a potent vasodilator and inhibitor of platelet aggregation, adhesion and proliferation of vascular smooth muscle cells, and it prevents the development of atherosclerosis [11]–[14]. Thus, in response to various neurohumoral stimuli, including 17ß-estradiol, endothelial cells release more NO, which produces vasodilatation and hyperpolarization of the vascular smooth muscle cells. In addition, this NO could also open K^+^ channels [15], [16], which contribute to maintain adequate vascular function.

K^+^ channel opening hyperpolarizes smooth muscle, which, by decreasing calcium entry through voltage-dependent Ca^2+^ channels, leads to vasodilatation [17]. Many subtypes of K^+^ channels have been identified in endothelial and smooth muscle cells: voltage-dependent K^+^ channel (*K_v_*), large (*BK_Ca_*), intermediate (*IK_Ca_*) and small (*SK_Ca_*) conductance Ca^2+^-activated K^+^ channels, ATP-sensitive K^+^ channels (*K_ATP_*), and inward rectifier K^+^ channels (*K_ir_*) [17]–[19]. The fundamental properties of these channels, as well as their responses to various stimuli including vasoconstrictors and vasodilators and their associated signal pathways have been described in several reports [18], [19]. Moreover, the involvement of K^+^ channels in cardiovascular disorders depends on the vascular tissue or species studied [18]. Thus, *BK_Ca_* channels play a key role in regulating vascular tone in resistance arteries [20], while the aortic tone is strongly dependent on the activity of *K_v_* channels [21].

The activation of Na^+^K^+^-ATPase activity is another important mechanism contributing to the maintenance of vascular tone and membrane potential of vascular smooth muscle cells [22], [23]. The Na^+^K^+^-ATPase [24] is an enzyme with gender-dependent function and expression [25]. This enzyme contributes to maintain the resting membrane potential, vascular tone and contractility regulation [22], [26], and it is influenced by endothelium-derived factors, shear stress and hormones [27], [28].

Although a variety of studies [1]–[4] have demonstrated significant male-female differences in vascular reactivity, the roles of K^+^ channels and Na^+^K^+^-ATPase activity interaction in these differences are still unknown. Therefore, the aim of this study was to evaluate gender differences in K^+^ channel subtypes and Na^+^K^+^-ATPase activity in male and female rat aorta. Our hypothesis is that the roles of K^+^ channels and Na^+^K^+^-ATPase activity might be influenced by gender because of nitrergic modulation and the influence of estrogen. For this, we investigated the difference of gender on: 1) participation of different subtypes K^+^ channels in the relaxation induced by acetylcholine; 2) Functional Na^+^K^+^-ATPase activity; 3) involvement of the NO pathway in Na^+^K^+^-ATPase functional activity. Our findings provide evidence that the K^+^ channels activation is different between genders and depends on *BK_Ca_* in females and *K_v_* in males while Na^+^K^+^-ATPase activity is greater in males.

## Materials and Methods

### Experimental Animals

Fifty five (55) Wistar rats that were 9±1 weeks old were used in this study (twenty five males with 268±4 g and thirty females with 271±5 g). The rats were housed at constant room temperature, humidity and light cycles (12-h light/dark), had free access to tap water, and were fed standard rat chow ad libitum. Female rats were studied using random selection regardless of the stage of the ovarian cycle. Since the ovarian cycle in rats is frequent (every 4 to 5 days) and the estrous stage is short (12 h), the average data from all female rats should cancel out the effects of possible fluctuations in sex hormone levels at specific stages of the ovarian cycle and should, roughly, represent the average changes in vascular reactivity during all stages of the ovarian cycle.

Care and use of laboratory animals were in accordance with NIH guidelines. All experiments were conducted in compliance with the guidelines for biomedical research, as stated by the Brazilian Societies of Experimental Biology, and were approved by the Institutional Ethics Committee (CEUA-EMESCAM 003/2007 and 004/2007).

### Vascular Reactivity Studies

Rats were anesthetized using urethane (1.2 g/Kg, i.p.) and sacrificed by exsanguination. The aorta was cleaned of fat and connective tissue and cut into four to five mm-long rings. Rings were mounted between parallel wires (thickness: 0.34 mm) in tissue baths (5 mL volume) containing Krebs-Henseleit solution (in mM: 124 NaCl, 4.6 KCl, 2.5 CaCl_2_, 1.2 MgSO_4_, 1.2 KH_2_PO_4_, 0.01 EDTA, 23 NaHCO_3_) and gassed with 95% O_2_ and 5% CO_2_ (pH 7.4) at 37°C. The K^+^-free solution was prepared by substituting KCl with NaCl and KH_2_PO_4_ with NaH_2_PO_4_ to maintain the osmolar concentration. Arterial segments were stretched to a resting tension of 1 g. Isometric tension was recorded using a force transducer (TSD 125 C, CA, USA) connected to an acquisition system (MP100A, BIOPAC System, Inc., Santa Barbara, USA).

After a 45 min equilibration period, all aortic rings were exposed twice to 75 mM KCl. The first exposure tests the functional integrity of vessels, and the second exposure assesses the maximum tension developed. Afterwards endothelial integrity was evaluated by administering ACh (10 µM) to a bath with aortic rings that were precontracted with phenylephrine (PHE, 0.1 mM). A relaxation equal to or greater than 90% was considered demonstrative of the functional integrity of the endothelium. To evaluate the role of NO relaxation was induced by ACh. After a 45-min washout period, aortic rings from male and female rats were pre-contracted with PHE (0.1 mM) and the concentration-response curves to ACh (0.1 nM – 300 µM) were determined. In sequence vessels were incubated with *N*^G^-nitro-L-arginine methyl ester (L-NAME, 100 µM) to investigate gender effects on NO production.

The K^+^ channel contribution to ACh-induced relaxation was assessed in aortas that were previously incubated for 30-min with the following K^+^ channel blockers: 2 mM tetraethylammonium (TEA), a nonselective K^+^ channel blocker; 5 mM 4-aminopyridine (4-AP), a selective voltage-dependent K^+^ channel blocker (*K_v_*); 30 nM iberiotoxin (IbTX), a selective *BK_Ca_* blocker; 0.1 µM charybdotoxin (ChTX), a nonspecific *K_Ca_* (*BK_Ca_,* and *IK_Ca_)* and *K_v_* (*K_v1.3_*) blocker; and 0.5 µM apamin, a selective small-conductance Ca^2+^-sensitive K^+^-channel blocker (*SK_Ca_*) [29].

In another set of experiments, the functional activity of the Na^+^K^+^-ATPase was measured in segments from female and male rats using K^+^-induced relaxation, as described by Webb and Bohr (1978) [30] and modified by Rossoni *et al.* [26]. After a 30-min equilibration period in normal Krebs, the preparations were incubated for 30 min in K^+^-free Krebs. The vessels were subsequently pre-contracted with PHE, and once a plateau was attained, the KCl concentration was increased stepwise (1, 2, 5 and 10 mM) with each step lasting for 2.5 min. To evaluate the contribution of the Na^+^K^+^-ATPase functional activity, after a washout period, the preparations were incubated with 100 µM ouabain (OUA) for 30 min to inhibit sodium pump activity, and the K^+^-induced relaxation curve was repeated. There was not any basal vascular contraction after incubation with OUA (data not shown). To study the involvement of nitric oxide in Na^+^K^+^-ATPase functional activity, the rings were incubated with L-NAME 100 µM.

### Statistical analyses

All values are expressed as the mean ± S.E.M. Contractile responses are expressed as a percentage of the maximum response induced by 75 mM KCl. K^+^-induced relaxation is expressed as a percentage of the tone previously obtained using PHE. The K^+^-induced relaxation curves were generated using nonlinear regression analysis of the concentration-response curves. ACh relaxation responses are expressed as a percentage of relaxation from the maximal contractile response. For each concentration-response curve, the maximal response (*R_max_*) and agonist concentration that produced 50% of the maximal response (*pEC_50_*,-log EC*_50_*) were calculated using non-linear regression analysis (GraphPad 5 Software, Inc., San Diego, CA). The agonist sensitivities are expressed as *pEC_50_*. Vasodilator responses are expressed as percentage of previous contraction. To compare the effect of drugs on ACh-induced responses in female and male rat aortic segments, certain results are expressed as differences in the area under the concentration–response curves (dAUC) between control and experimental conditions. AUCs were calculated from individual concentration–response curve plots. The differences are expressed as percentage of the control AUC. Differences were analyzed using the Student’s *t*-test and either a one or two-way ANOVA followed by a Bonferroni test. *P<*0.05 was considered significant.

### Drugs and reagents

l-Phenylephrine hydrochloride, ACh chloride, SNP, urethane, OUA, L-NAME, TEA, 4-AP, IbTX, ChTX and apamin were purchased from Sigma-Aldrich (St. Louis, USA). The salts and reagents used were of analytical grade from Sigma-Aldrich and Merck (Darmstadt, Germany).

## Results

### Gender differences in ACh-induced concentration-dependent relaxation

**Figure 1:**
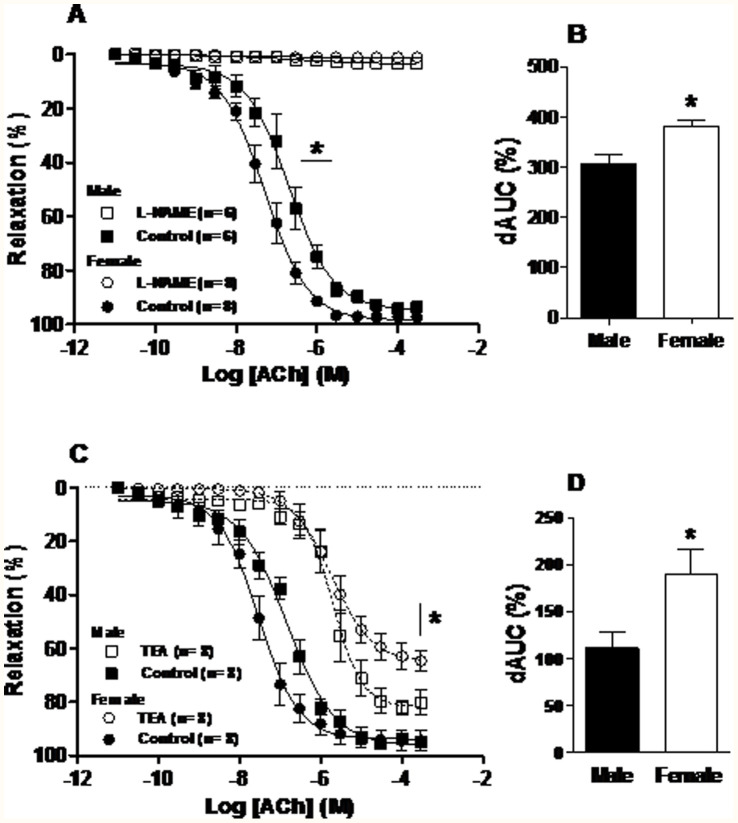
Acetylcholine (ACh) concentration-response curve for the aortic rings from male and female rats. Endothelium intact (Control) and N_G_-nitro-L-arginine methyl ester (L-NAME 100 µM) curves (A); Difference of the area under curve (dAUC) control and L-NAME (B); Control and tetraethylammonium (TEA 2 mM) curves (C); dAUC control and TEA (D). *P<0.05, *pEC_50_* male *vs.* female control; and *R_max_* male vs. female TEA *P<0.05, dAUC male *vs.* female. Student’s *t*-test. Number of animals used is indicated in parentheses.

The maximum response to ACh was similar between male and female groups, but sensitivity was greater in females compared with the male group (Figure 1A, Table 1). As expected, incubation with 100 µM L-NAME similarly inhibited ACh-induced relaxation in males and females (Figure 1A). Figure 1B demonstrates that, after incubation with L-NAME the dAUC was greater in the female group (Male: 304±19%, n = 6; Female: 380±14%, n = 8, P<0.05). This result suggests that NO had a greater influence on the functional ACh-induced relaxation in the female group compared with the male group.

**Table 1:** Parameters from the maximum response (*R_max_*) and agonist concentration that produced 50% of the maximum response (*EC_50_*) for the ACh concentration-response curve in aortic rings from male and female rats in an intact endothelium (Control) and incubated with tetraethylammonium (TEA), aminopyridine (4-AP), iberiotoxin (IbTX), charybdotoxin (ChTX) and apamin.

	*pEC_50_*		*R_max_*	
	Male	female	male	female
**Control**	6.75±0.08 (n = 17)	7.15±0.11* (n = 25)	95.30±1.12 (n = 17)	95.65±1.97 (n = 25)
**L-NAME**	6.92±0.91 (n = 6)	8.75±0.91 (n = 8)	3.47±1.26 (n = 6)	1.04±0.51 (n = 8)
**TEA**	5.74±0.14 (n = 8)	5.79±0.21 (n = 8)	80.68±3.84 (n = 8)	64.24±4.71† (n = 8)
**4-AP**	5.01±0.18 (n = 7)	5.50±0.14* (n = 11)	49.86±5.21 (n = 7)	84.26±3.85† (n = 11)
**IbTX**	6.50±0.14 (n = 13)	6.57±0.18 (n = 12)	97.86±2.41 (n = 13)	83.59±4.17† (n = 12)
**ChTX**	6.61±0.31 (n = 14)	6.37±0.20 (n = 13)	96.50±2.61 (n = 14)	75.59±4.49† (n = 13)
**Apamin**	7.03±0.17 (n = 7)	6.86±0.22 (n = 11)	93.84±2.01 (n = 7)	73.61±4.91† (n = 11)

Results are expressed as the mean ± SEM; maximal effect (*R_max_*); -log one-half *R_max_ (pEC_50_*); male and female intact endothelium (Control); tetraethylammonium (TEA); 4-aminopyridine(4-AP); iberiotoxin (IbTX); charybdotoxin (ChTX); apamin; and *N*^G^-nitro-L-arginine methyl ester (L-NAME). *P<0.05 (*pEC_50_* of female *vs.* male rats) and †P<0.05 (*R_max_* of female *vs.* male rats). Results are expressed as the mean ± S.E.M. Differences were analyzed using Student’s *t*-test, and P<0.05 was considered significant.

To investigate the role of K^+^ channels TEA was used. Figure 1C shows the results obtained during incubation with TEA, a nonselective K^+^-channel blocker. *R_max_* was reduced in both groups (Table 1). However, the inhibitory effect of TEA on the ACh-induced relaxation was greater in the female group (Figure 1D).

**Figure 2:**
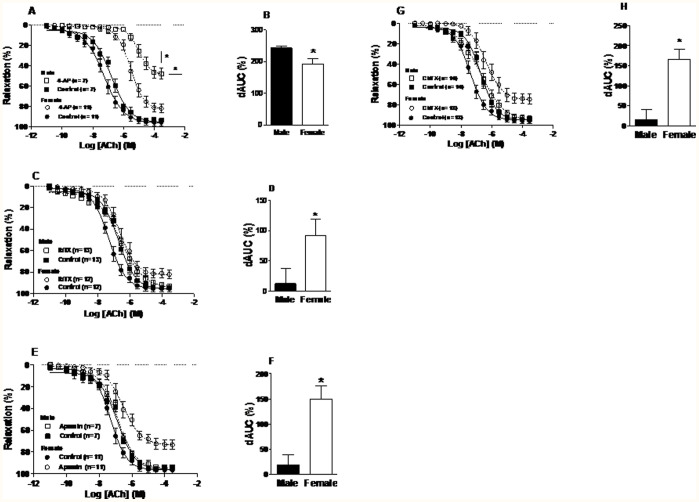
Acetylcholine (ACh) concentration-response curve for the aortic rings from male and female rats. Endothelium intact (Control) and 4-aminopyridine (4-AP 5 mM) curves (A); Difference of the area under curve (dAUC) control and 4-AP (B); Control and iberiotoxin (IbTX 30 nM) curves (C); dAUC control and IbTX (D); Control and apamin (0.5 µM) curves (E); dAUC control and apamin (F) and Control and charybdotoxin (ChTX 0.1 µM) curves (G); dAUC control and ChTX (H). *R_max_* *P<0.05, male vs. female 4-AP, IbTX, Apamin and ChTX incubations. *P<0.05, dAUC male vs. female. Student’s *t*-test. Number of animals used is indicated in parentheses.

In the presence of 4-AP, a specific voltage-dependent K^+^ channel inhibitor, ACh-dependent relaxation was reduced in both groups. However, males were more sensitive and had a smaller *R_max_* than the female group (Figure 2A, see Table 1). Figure 2B shows that the dAUC was greater in males, suggesting that *K_v_* participates more in the ACh-dependent relaxation of this group.

To evaluate the role of calcium-activated K^+^ channels, aortic rings were incubated with the selective blockers, IbTX *(BK_Ca_* blocker) (Figure 2C, Table 1) and Apamin (*SK_Ca_* blocker) (Figure 2E, Table 1), and the nonspecific blockers, ChTX (*K_Ca_* and *K_v_* blocker) (Figure 2G, Table 1). The three calcium-activated K^+^-channel inhibitors reduced *R_max_* more in females compared with males. The dAUC after incubation with three calcium-activated K^+^-channel inhibitors was greater in female compared with the male group (Figure 2D, F and H).

### Gender differences in functional Na^+^K^+^-ATPase activity

**Figure 3:**
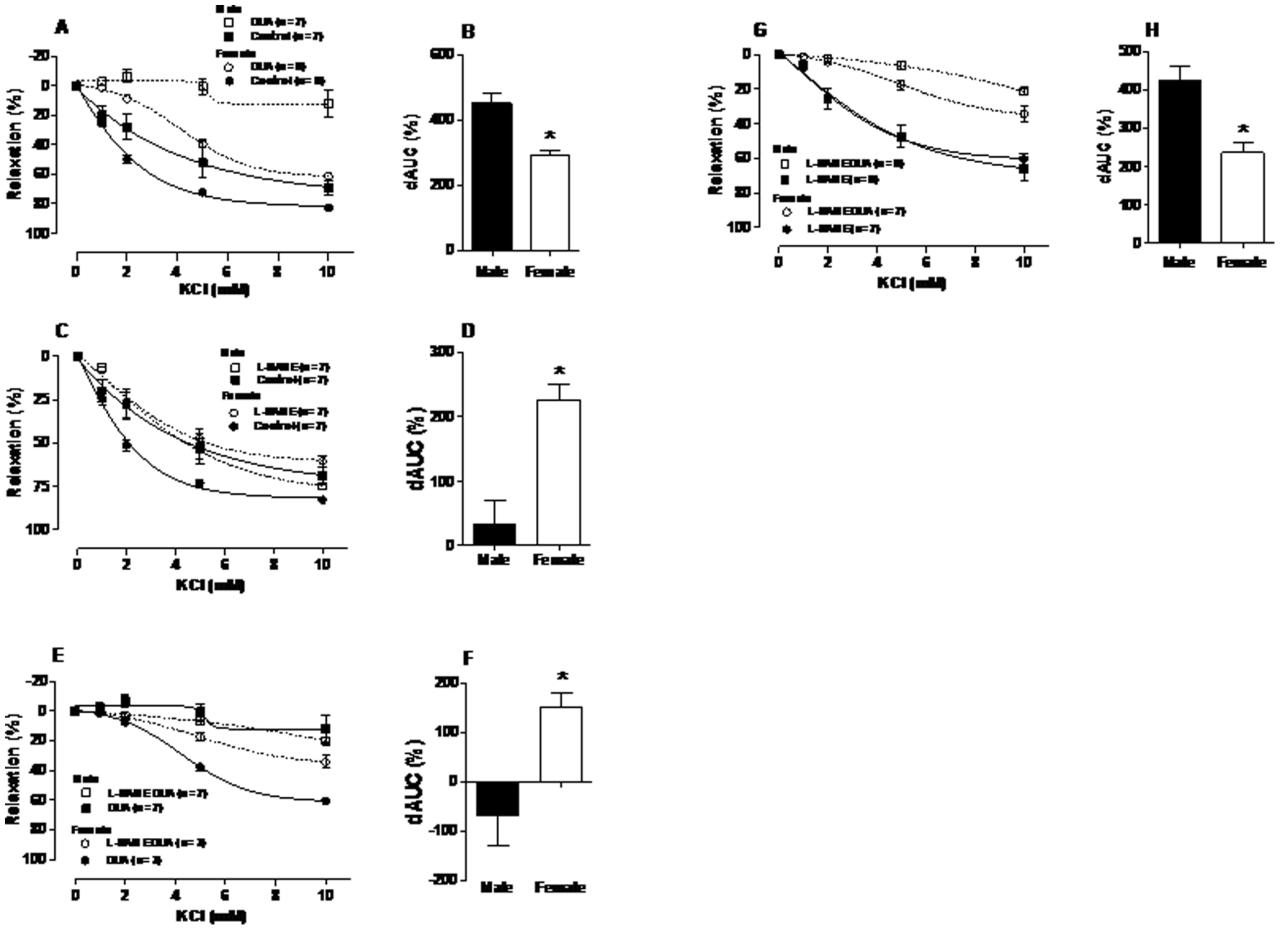
K^+^-induced relaxation in aortic rings from males and females rats after incubation in a K^+^-free medium and contracted using phenylephrine (PHE) in an intact endothelium (Control), incubated with ouabain (OUA 100 µM), and incubated with L-NAME (100 µM): Control and OUA curves (A); Difference of the area under curve (dAUC) control and OUA (B); Control and L-NAME curves (C); dAUC control and L-NAME (D); OUA and L-NAME plus OUA curves (E); dAUC OUA and L-NAME plus OUA (F); L-NAME and L-NAME plus OUA curves (G); dAUC L-NAME and L-NAME plus OUA (H). ^*^P<0.05, male *vs*. female using Student’s *t*-test. Number of animals used is indicated in parentheses.

The functional Na^+^K^+^-ATPase activity, as evaluated by K^+^-induced relaxation in aortic rings with an intact endothelium from male and female groups. The K^+^-induced relaxation was greater in females compared with the male group (Figure 3A, Table 1). Previous studies showed that OUA inhibits the Na^+^K^+^-ATPase [22], [27] and also induces an intracellular increase in Na^+^ and Ca^2+^ concentrations via Na^+^/Ca^2+^-exchanger inhibition and leads to an increment in vascular tone [22], [31]. As expected, Figure 3A demonstrates that, after incubation with 100 µM OUA, K^+^-induced relaxation was reduced in both groups. However, this reduction was greater in the male than female group. The difference between groups of the functional Na^+^K^+^-ATPase activity in K^+^-induced relaxation was studied evaluating the differences in the dAUC with and without OUA. The dAUC was greater in male compared to female (Male: 451±32%, n = 7; Female: 291±15* %, n = 8; *P<0.05), suggesting that functional Na^+^K^+^-ATPase activity is greater in males than in females (Figure 3B).

Figure 3C demonstrates that, after incubation with L-NAME, K^+^-induced relaxation was reduced only in the female group (P<0.05). The dAUC was greater in the female group (Male: 43.70±59.53%, n = 7; Female: 207±34* %, n = 7; *P<0.05) (Figure 3D), suggesting a great NO modulation for this group. To verify the NO participation in OUA-mediated inhibition of K^+^-induced relaxation, the rings were superfused in a solution with OUA plus L-NAME (Figure 3E). As expected, OUA reduced K^+^-induced relaxation in both groups. However, in the male group, there was no difference between K^+^-induced relaxation after incubation with OUA or OUA plus L-NAME. In contrast, in the female group, this difference was evident, as demonstrated by the dAUCs (Male: – 67.86±61.65%, n = 9; Female: 152±27* %, n = 7; *P<0.05) (Figure 3F). This result suggests that NO might have a greater influence on functional Na^+^K^+^-ATPase activity in the K^+^-induced relaxation in female group, but not in males. To evaluate the participation of the functional Na^+^K^+^-ATPase activity in K^+^-induced relaxation without NO, we compared the curves obtained during incubation with L-NAME with and without OUA (Figure 3G). K^+^-induced relaxation was reduced in both groups after OUA and L-NAME incubation (P<0.05). However, the dAUC was smaller in the female group (Male: 424±35%, n = 7; Female: 235±24* %, n = 7; *P<0.05) (Figure 3H).

## Discussion

The major finding from this study indicates that the involvement of K^+^ channels in ACh-induced relaxation differs between genders being dependent on *BK_Ca_* in females and *K_v_* in males. We also demonstrated that functional Na^+^K^+^-ATPase activity in the vascular K^+^-induced relaxation is greater in males than in females.

In this study we investigated the NO participation on ACh-induced relaxation in the isolated rings of aortas from male and female rats. As expected, our results demonstrated that NO bioavailability was greater in the female group. Considering female rats were studied independent on estrous cycle, it is not possible to correlate these responses to a specific hormone. However, considering that ACh-induced vasorelaxation is not affected by the day of oestrous cycle in perfused mesenteric vascular beds due to the small contribution of NO in this vessel [32], it is feasible that this result could depend on, at least in part, by the rapid non-genomic effect of estrogen mediating vasodilatation via activation of endothelium-dependent vascular relaxation pathways [9], [33]–[35]. On the other hand, Della Lucca et al. [33] demonstrated that the responses to cirazoline and ACh in the uterine vasculature of virgin rats were cycle day-dependent, suggesting that there are discrepancies about the effect of the ovarian cycle on vasomotor responses. Nevertheless, the participation of other female hormones cannot be discarded and need to be investigated.

To evaluate whether K^+^ channels contribute to the vascular relaxation differences in males and females, ACh-induced relaxation was assessed in aortas after incubation with K^+^ channel blockers. In the presence of TEA, a nonselective K^+^ channel blocker, our results demonstrated that K^+^ channels contribute more to ACh-induced relaxation in females than in males. Although, in the present study, is not possible to attribute these responses to any specific female hormone, it is well know that estrogen, the main ovarian hormone, can affect vascular smooth muscle relaxation via a direct effect on K^+^ channel activation [36]–[38].

The *BK_Ca_* and *K_v_* channels are the primary ion-conducting pathways that regulate resting membrane potential and vascular tone [17]. However, the observation of K^+^ currents by patch-clamp in single myocytes showed that *K_v_* currents generate more negative potential than *BK_Ca_* currents. This result suggests that *K_v_* contributes more to the control of resting membrane potential in small blood vessels [39] and rat aortic smooth muscle cells [21]. To evaluate *K_v_* participation in the gender difference on the ACh-induced relaxation, we used 4-aminopyridine, a selective inhibitor of this channel. Our results showed that the contribution of K_v_ channel on the ACh-induced relaxation is greater in male than in female animals.

To evaluate the roles of *BK_Ca_* and *SK_Ca_*, we used the channel blocker IbTX and apamin, respectively. Our results showed the contribution of *BK_Ca_* and *SK_Ca_* channel on the ACh-induced relaxation is greater in female than in male animals. Similar to our results, Yang et al. [36] also demonstrate that the *BK_Ca_* current in coronary smooth muscle was greater in female than in male animals.

When we used ChTX, a nonspecific *K_Ca_* and *K_v_* blocker, the relaxation to ACh was smaller in females than males. These results corroborate the finding using TEA, confirming the higher influence of K channels in the female than in male. However, different from TEA, ChTX inhibits *BK_Ca_, IK_Ca_* and *K_v1.3_* isoforms [29], [40] and it is unable to inhibit *K_v2.1_,* which plays a predominant role in aortic smooth muscle [21]. Moreover, ChTX inhibits *IK_Ca_* channels more specificity than *BK_Ca_* channels [40]. The inhibition of *IK_Ca_*, prevent the hyperpolarization of both the endothelial and the smooth muscle cells.

In fact, it has been demonstrated that estrogen is involved in activation of endothelial receptors that stimulate the *K_Ca_* channel to hyperpolarize the endothelial and vascular smooth muscle cells [37]. It is possible that inhibition of the *K_Ca_* channels impairs more the relaxation in females than in males.

The vascular Na^+^K^+^-ATPase activity is another important mechanism responsible for maintaining the cellular membrane potential and contributes to the regulation of vascular tone and blood pressure [23]. Therefore, in the presence of ouabain (100 µM), gender-dependent functional Na^+^K^+^-ATPase activity was evaluated during its inhibition by external K^+^ withdrawal. This procedure is known to induce a gradual cell depolarization that is reverted by K^+^ reintroduction leading to a hyperpolarization. Our results demonstrated that the vascular Na^+^K^+^-ATPase functional activity is higher in male than in female rats. Palacios et al. [25] demonstrated that female rat aorta has smaller levels of the Na^+^K^+^-ATPase α_1_ isoform and greater α2 isoform compared with male rats. In fact, it has been proposed that α1, but not α2 or α3 isoforms, is involved in ACh-mediated hyperpolarization in rat aortic endothelium [41] and in porcine aortic [42] and human umbilical vein [43] endothelial cells. These results are also in accordance to the findings that the endothelium of large vessels predominantly expresses the α1 isoform of Na^+^K^+^-ATPase [44].

Palacios et al. [25] found that the incubation of arterial smooth muscle with ACh significantly increased ouabain-sensitive 86Rb/K uptake in the female rat aorta. The increase in Na^+^K^+^-ATPase activity in response to ACh was only observed in intact arteries, suggesting a direct influence of an endothelial factor. Although these results seem contradictory to our results is important to emphasize that in our study we use a different technique to evaluate vascular ouabain sensitive Na^+^K^+^-ATPase functional activity. The potassium-induced relaxation is a protocol used by authors in the literature in order to evaluate the functional ouabain sensitive Na^+^K^+^-ATPase activity. [26], [29], [45]. Therefore, the objective of performing the relaxation K^+^-induced and not the ACh-induced relaxation protocol was specifically to assess the contribution of the ouabain sensitive Na^+^K^+^-ATPase activity in vascular relaxation similarly to study of Fiorim et al. [45] conducted in our laboratory. The used of OUA in the curve of ACh could demonstrate the role of Na^+^K^+^-ATPase in relaxation endothelium-dependent, but results of this protocol must be carefully assessed because in some arteries the Na^+^K^+^-ATPase is a target for K^+^ acting as an endothelium-derived hyperpolarizing factor (EDHF) [46]. Furthermore, the K^+^-induced relaxation solution used in this study, is potassium free. Skaug and Detar [47] report changes in K^+^ channels behavior when an extracellular K^+^concentration is modify, which might compromise their influence on the Na^+^K^+^-ATPase activity.

Several studies demonstrated that NO is an important hyperpolarizing factor in conductance arteries [15], [16]. Therefore, in order to understand the influence of NO on the vascular functional Na^+^K^+^-ATPase activity, we used the non-specific NOS inhibitor L-NAME. The results demonstrated that the K^+^-induced relaxation was reduced only in the female group suggesting that basal NO modulation of the K^+^-induced relaxation is greater in the female group. Our results are similar to previous studies indicating enhanced basal NO production in female rats [48], [49]. The results suggest that the NO influence on the vascular Na^+^K^+^-ATPase activity might be higher in the female group than in males. The results presented in figures 3C and 3D demonstrated that in the presence of L-NAME, K^+^-induced relaxation was reduced only in the female group, suggesting a gender dependence on NO synthesis, as observed during ACh-induced relaxation. Thus, it seems that, although the functional vascular Na^+^K^+^-ATPase activity is greater in male, the nitrergic modulation of K^+^-induced relaxation is higher in the female group. Similarly, results presented in the figures 3E and 3F (OUA, L-NAME plus OUA curves and dAUC), reinforces a higher NO influence on functional Na^+^K^+^-ATPase activity in the K^+^-induced relaxation in female group. Results presented in the figures 3G and 3H (L-NAME, L-NAME plus OUA curves and dAUC), confirmed that, in the absence of NO, the K^+^-induced relaxation is similar in male and female. Moreover, the relaxation during Na^+^K^+^-ATPase inhibition, in the absence of NO, was more reduced in male then in female group, corroborating the conclusion that functional Na^+^K^+^-ATPase activity in the K^+^-induced relaxation seems to be greater in male than in female.

Taking into account all the results presented here, it is tempting to say that, the important gender difference relies upon the mechanisms involved in the regulation of the vascular tonus. It seems that K^+^-induced relaxation in male rats depends mainly on functional Na^+^K^+^-ATPase activity while *K_Ca_* channels and NO have more influence in female rats.

Limitations to the present investigation need to be addressed. First, the effects of gender on the vascular ion channels should also be analyzed using patch-clamp to study the electrophysiological properties of K^+^ channels. Secondly, in the present study, aortic female rats were studied without correlation with estrous cycle, being impossible to attribute the vascular response to any specific female hormone. Other studies are necessary to understand the specific contribution of sex hormones on observed changes.

In conclusion, our results demonstrated that ACh-induced relaxation involves different mechanisms in male and female rats. The ACh-induced relaxation has a greater participation of *K_Ca_* in female and *K_v_* in males. Also, the vascular K^+^-induced relaxation has a higher participation of Na^+^K^+^-ATPase in male than in female and NO participates more modulating the functional Na^+^K^+^-ATPase activity in the female group.
